# Muscle strength and gait speed rather than lean mass are better indicators for poor cognitive function in older men

**DOI:** 10.1038/s41598-020-67251-8

**Published:** 2020-06-25

**Authors:** Sophia X. Sui, Kara L. Holloway-Kew, Natalie K. Hyde, Lana J. Williams, Sarah Leach, Julie A. Pasco

**Affiliations:** 1 Deakin University, Geelong, VIC Australia; 2 GMHBA, Geelong, VIC Australia; 3 Department of Medicine–Western Campus, The University of Melbourne, St Albans, VIC Australia; 4 Department of Epidemiology and Preventive Medicine, Monash University, Melbourne, VIC Australia; 5 University Hospital Geelong, Barwon Health, Geelong, VIC Australia

**Keywords:** Epidemiology, Risk factors, Dementia, Neuromuscular disease

## Abstract

We aimed to examine muscle strength, function and mass in relation to cognition in older men. This cross-sectional data-set included 292 men aged ≥60 yr. Handgrip strength (kg) was measured by dynamometry, gait speed by 4-metre walk (m/s) and appendicular lean mass (kg) by dual-energy x-ray absorptiometry. Cognition was assessed across four domains: psychomotor function, attention, visual learning and working memory. Composite scores for overall cognition were calculated. Bivariate analyses indicated that handgrip strength and gait speed were positively associated with cognitive function. After accounting for confounders, positive associations between individual muscle (or physical) measures and cognitive performance were sustained for handgrip strength and psychomotor function, gait speed and psychomotor function, gait speed and attention, handgrip strength and overall cognition, and gait speed and overall cognition. In multivariable models, handgrip strength and gait speed independently predicted psychomotor function and overall cognition. No associations were detected between lean mass and cognition after adjusting for confounders. Thus, low muscle strength and slower gait speed, rather than low lean mass, were associated with poor cognition in older men.

## Introduction

The proportion of elderly people in Australia has been increasing and will continue to increase in coming decades^1^. Investigations into factors that influence older Australians’ health make significant contributions to enabling them to live independently and maintain a high quality of life^2^. Dementia, a decline in mental ability due to a wide range of progressive and acquired neurocognitive disorders, is a major health threat to older people and carries a substantial social and economic burden^3^. Mild cognitive impairment (MCI), a pre-dementia stage, can be identified years before the onset of established dementia. Currently, there is no effective psychological or medical treatment for dementia; however, MCI may be preventable and reversible by addressing modifiable risk factors^4^.

Previous research has demonstrated that poor skeletal muscle health is associated with an increased likelihood of MCI and other adverse physical and mental outcomes^5–11^. Sarcopenia is characterised as age-associated low muscle mass in conjunction with low muscle strength or physical performance^12–16^. A systematic review and meta-analysis published in 2016 revealed that sarcopenia was associated with cognitive impairment^17^, and when assessed separately, muscle strength^18^, gait^19^ and gait speed^20,21^ were identified in association with cognitive function. A non-linear relationship between muscle mass and strength^22,23^ raises uncertainty about the roles of the components of sarcopenia in the relationship between sarcopenia and cognition. In many studies, general cognitive function has been assessed using global cognitive tests, such as The Mini-Mental State Examination (MMSE) and its modified versions, and these tests have limited ability to identify subtle differences in cognitive deficits^24^. To date, few studies have examined the relationship between the components of sarcopenia and the specific domains of cognitive function.

Demographics, health behaviours, and life experiences can affect both physical and mental health and should be considered as potential confounders in associations between muscle health and cognitive function^25–30^. Thus, the aim of our study was to determine whether muscle strength, performance and mass in older men are associated with cognitive function, both overall and across specific domains.

## Methods

### Study design

This cross-sectional study is part of the Geelong Osteoporosis Study (GOS), an ongoing longitudinal population-based study. Briefly, age-stratified samples of men and women were randomly selected from electoral rolls for the Barwon Statistical Division in south-eastern Australia. For the male cohort, baseline data for 1,540 men were collected 2001–2006 (67% response), and again at 5-, 6- and 15-year follow-up phases. Details of the GOS have been published elsewhere^31^. This study utilises data from the 15-year follow-up phase for men. Comparable data are being collected for female participants.

### Participants

The current analysis involved 292 men aged 60–96 years assessed in the most recent follow-up phase (2016–2019). Participants completed a series of questionnaires regarding lifestyle and demographic characteristics in conjunction with physical and mental health assessments. Participants were mostly Caucasian (~98%). All participants provided written, informed consent to participate in the study. A listing on the Commonwealth electoral roll as a resident of the Barwon Statistical Division was the inclusion criterion; individuals who had resided in the region for less than 6 months and those unable to provide informed, written consent met exclusion criteria^31^. Thus, individuals with severe cognitive impairment or dementia and were not able to provide consent were excluded from the study. The study was approved by the Human Research Ethics Committee at Barwon Health.

### Measures

#### Cognitive function

Cognitive function was assessed using the CogState Brief Battery (CBB), a computer-based neuropsychology battery^32^, described in detail elsewhere^5,33–35^. In brief, CBB involves responding to stimuli cards as part of a detection task (DET), an identification task (IDN), a one-card learning task (OCL) and a one-back task (OBK). These four tasks assess the cognitive domains of psychomotor function, visual attention, recognition memory/learning and working memory, respectively. Each task included a practice trial and a real test. Participants completed the tasks in a quiet room accompanied by a researcher. As stated in the guidelines, the aim of the tasks was to capture performance, in terms of both speed and accuracy^12^, in each cognitive domain. In DET, IDN, and OBK tasks, the primary measurement scores, named “lmn” (unit: log_10_ million seconds), were calculated according to speed in supplying correct answers (mean of log_10_-transformed reaction times), so lower scores indicated better performance. The primary measurement scores in the OLC task, labelled “acc” (unit: arcsine square root proportion correct) were calculated according to accuracy (arcsine transformation of the square root of the proportion of correct response), so higher scores indicated better performance. Scores for overall cognitive function (OCF) (unitless) were calculated by compositing the primary measures in the four cognitive domains^36^; higher scores indicated better performance. The primary outcomes for each task and composite measures were used for analyses. Administration of the CBB took about 20 minutes per participant; a previous study has demonstrated the validity and reliability of the CBB^33^.

#### Muscle parameters

Handgrip strength (HGS) was measured using an electronic handheld dynamometer (Vernier, LoggerPro3). The participant was seated in a comfortable position with the arm holding the dynamometer flexed at the elbow to 90 degrees. The participant squeezed the device using each hand three times with maximum effort for three seconds with a five-second interval between trials. The maximum value for each hand was used to calculate a mean HGS, which was used for all analyses. Lean soft tissue mass, a proxy measure for muscle mass, was assessed by whole body dual-energy X-ray absorptiometry (DXA; Lunar Prodigy-Pro, Madison, WI, USA). DXA-derived lean mass comprises non-fat and non-bone tissue and is correlated with skeletal muscle mass measured with magnetic resonance imaging^37^. Appendicular lean mass (ALM) was calculated as the sum of lean mass for the arms and legs and expressed relative to the square of height ALM/height^2^ (kg/m^2^). Short-term precision (calculated as the coefficient of variation on repeated whole body scans) was 0.9% for ALM. Usual gait speed^12,13,38^ was determined by measuring how many seconds the participant took to walk a distance of 4 metres and recorded as m/s; participants wore shoes and were asked to walk at their normal (preferred) walking speed, and used a walking aid if necessary.

#### Other measures

Weight and height were measured to the nearest ±0.1 kg and ±0.001 m. Body mass index (BMI) was calculated as weight/height^2^ (kg/m^2^). BMI < 18.5 kg/m^2^ was classified as underweight, 18.5–24.9 kg/m^2^ as normal weight, 25.0–29.9 kg/m^2^ as overweight, and ≥ 30 kg/m^2^ as obese^14,39^. Details about education, smoking status, marital status and mobility were obtained by self-report. Education was classified as secondary education completed (13-years of school education) or not, marital status as living with a partner or not, and mobility as active (if vigorous or light exercise was performed regularly) or sedentary^31^. In this study, active is equivalent to “moves, walks and works energetically and participate in vigorous exercise (very active); or walks at brisk pace, does normal housework or other work. Engages in light exercise (active)”. Participants who smoked at least one cigarette per day were classified as current smokers, otherwise as non-smokers.

### Statistical analysis

After checking the data for normality using histograms, inter-group differences were examined using one-way analysis of variance or Mann–Whitney tests for continuous variables; chi-squared tests or Fisher exact test were employed for categorical variables. Linear regression models were used to investigate associations between muscle parameters (HGS for strength, gait speed for physical performance and ALM/height^2^ for lean mass) and cognitive function. The cognitive function scores on four tasks (DET, IDN, OCL, and OBK) and OCF were included as separate dependent variables. For each muscle parameter, regression analyses included an unadjusted model (model 1), an age-adjusted model (model 2), and a model that also considered potential confounders that were identified (in a stepwise sequence) in the order of education status, marital status, mobility, smoking status and BMI; confounding variables were retained if p < 0.05 (model 3). BMI was not included in the model for ALM/height^2^, to avoid collinearity. Finally, multivariable models were developed for each cognitive parameter by considering independent contributions of muscle strength, physical performance and lean mass (and confounding variables) in the same model (model 4). A sensitivity analysis excluded outliers, whose extreme cognitive scores likely reflected inconsistencies in cognitive performance tests, with scores of two standard deviations beyond the mean in specific cognitive domains. All the analyses were conducted using IBM SPSS (v24, USA) and Minitab (v18, USA).

## Results

### Participant characteristics

Demographic and anthropometric characteristics for participants are presented in Table 1. The mean BMI was in the overweight range, few participants smoked, and nearly three-quarters had completed secondary education. Most participants lived with a partner and two-thirds were physically active.

**Table 1:** Participant characteristics. Data (n = 292) are presented as mean (±SD), n (%) or median (IQR).

Demographic characteristics	Age (yr)	70 (66–77)
	Height (m)	1.73 (±0.07)
	Weight (kg)	84.1 (±13.8)
	BMI (kg/m^2^)	28 (±4)
	Current smoker	9 (3%)
	Education status (completed year 12)	204 (70%)
	Marital status (living with a partner)	239 (82%)
	Mobility (active)	195 (67%)
Muscle strength	HGS (kg)	21 (±7)
Muscle mass	ALM (kg)	24.7 (±3.3)
	ALM/height^2^ (kg/m^2^)	8.3 (±0.9)
Physcial performance	Gait speed (m/s)	0.9 (±0.2)
Cognitive function	DET	2.48 (±0.11)
	IDN	2.68 (±0.08)
	OCL	0.93 (±0.10)
	OBK	2.91 (±0.11)
	OCF	0.001(±0.72)

HGS: handgrip strength (kg); ALM: relative appendicular lean mass (kg/m^2^); DET: the detection task (log10 milliseconds) measuring psychomotor function; IDN: the identification task (log10 milliseconds) measuring attention; OCL: the one-card learning task (arcsine square root proportion correct) measuring memory/learning; OBK: one-back task (log10 milliseconds) measuring working memory; OCF: overall cognitive function (unitless).

There was an age-related decline in cognitive performance according to scores for DET, IDN, OBK, OCL, and OCF (coefficient (B) = +0.004, +0.003, +0.005, −0.003, −0.04, respectively; all p < 0.001) and in HGS, ALM/height^2^ and gait speed (B = −0.39, −0.04, −0.01, respectively; all p < 0.001). Participants with completed secondary education had greater HGS and faster gait speed, and performed better on all cognitive domains and OCF (Table 2). Those living with a partner performed better on OCF and all cognitive domains expect OCL, and had higher values of HGS. The physically active group performed better on OCF and all cognitive domains except OCL, and had better values for all the muscle parameters. Smokers performed better on IDN, but had lower values for ALM/height^2^; no other differences between smokers and non-smokers were detected.

**Table 2:** Inter-group differences in parameters of cognitive function and muscle, assessed using Student’s t-tests. Data are shown as mean (±SD).

Groups	Education		p	Marital status		p	Mobility		p	Smoking status		p
	Completed secondary education	Not completed secondary education		Living with a partner	Living alone		Physically active	Sedentary		Current smokers	Non-smokers	
n	204	86		239	52		195	96		9	277	
***Cognitive function***												
DET	2.47 (±0.10)	2.51 (±0.13)	0.003	2.47 (±0.10)	2.51 (±0.14)	0.04	2.51 (±0.12)	2.46 (±0.10)	<0.001	2.48 (±0.09)	2.48 (±0.11)	0.90
IDN	2.67 (±0.07)	2.71 (±0.07)	0.001	2.67 (±0.07)	2.72 (±0.09)	0.003	2.67 (±0.07)	2.71 (±0.09)	<0.001	2.63 (±0.05)	2.68 (±0.08)	0.02
OCL	0.94 (±0.10)	0.90 (±0.10)	0.007	0.93 (±0.10)	0.93 (±0.10)	0.97	0.94 (±0.09)	0.92 (±0.11)	0.08	0.92 (±0.09)	0.93 (±0.10)	0.68
OBK	2.89 (±0.10)	2.94 (±0.10)	<0.001	2.90 (±0.10)	2.95 (±0.11)	0.002	2.89 (±0.10)	2.93 (±0.12)	0.002	2.91 (±0.09)	2.91 (±0.11)	0.86
OCF	0.12 (±0.68)	−0.29 (±0.72)	<0.001	0.06 (±0.67)	−0.30 (±0.84)	0.006	0.13 (±0.63)	−0.28 (±0.81)	<0.001	0.16 (±0.45)	−0.004 (±0.73)	0.34
***Muscle parameters***												
HGS	22 (±7)	19 (±7)	<0.001	22 (±7)	19 (±7)	0.002	23 (±7)	18 (±7)	<0.001	20 (±5)	21 (±7)	0.34
ALM/height^2^	8.3 (±0.9)	8.1 (±1.0)	0.07	8.3 (±1.0)	8.0 (±1.0)	0.12	8.4 (±0.9)	8.0 (±0.9)	<0.001	7.6 (±0.7)	8.3 (±0.9)	0.02
GS	0.9 (±0.2)	0.8 (±0.2)	0.001	0.9 (±0.2)	0.8 (±0.2)	0.13	0.9 (±0.2)	0.8 (±0.2)	<0.001	0.8 (±0.2)	0.9 (±0.2)	0.20

HGS: handgrip strength (kg); ALM: relative appendicular lean mass (kg/m^2^); GS: gait speed, measured as walking speed over 4 meters (m/s); DET: the detection task (log10 milliseconds) measuring psychomotor function; IDN: the identification task (log10 milliseconds) measuring attention; OCL: the one-card learning task (arcsine square root proportion correct) measuring memory/learning; OBK: one-back task (log10 milliseconds) measuring working memory; OCF: overall cognitive function (unit less).

### Multivariable analysis

HGS, ALM/height^2^ and gait speed were all positively associated with better performance on DET and OCF (model 1). After adjusting for age, the associations of ALM/height^2^ with DET and OCF were attenuated, but the associations of HGS and gait speed with DET and OCF were sustained (model 2). The best fit models (model 3) showed that, for every 1.0 kg increase in HGS, there was a 0.004 score (log_10_ million seconds) decrease in DET after adjusting for age, and a 0.02 score (unitless) increase in OCF after adjusting for age, education, and mobility. For every 1.0 m/s increase in gait speed, there was a 0.11 score decrease in DET after adjusting for age and mobility and a 0.52 score increase in OCF after adjusting for age, education and mobility (Table 3). Model 4 included muscle strength and physical performance together, and revealed that HGS and gait speed independently predicted DET, and no confounders were identified; HGS and gait speed independently predicted OCF before and after adjusting for age and education (Table 4).

**Table 3:** Linear regression analysis for predicting cognitive function in association with muscle parameters.

Outcomes	Exposures	Coefficient B (95%CI)	P value	Adj. R^2^	Coefficient B (95%CI)	P value	Adj. R^2^	Coefficient B (95%CI)	P value	Adj. R^2^
		Unadjusted (model 1)			Age adjusted (model 2)			Final (model 3)		
*DET*	HGS	−0.005 (−0.006, −0.003)	<0.001	0.10	−0.004 (−0.005, −0.002)	<0.001	0.12	−0.004 (−0.005, −0.002) ^a^	<0.001	0.12
	ALM/height^2^	−0.02 (−0.03, −0.003)	0.02	0.02	−0.007 (−0.02, 0.007)	0.33	0.08	—	—	—
	Gait speed	−0.16 (−0.23, −0.10)	<0.001	0.08	−0.12 (−0.19, −0.06)	<0.001	0.11	−0.11 (−0.18, −0.04) ^a^	0.003	0.12
*IDN*	HGS	−0.003 (−0.004, −0.002)	<0.001	0.07	−0.002 (−0.003, 0)	0.01	0.12	−0.001 (−0.002,0) ^*^	0.06	0.19
	ALM/height^2^	−0.02 (−0.03, −0.006)	0.001	0.03	−0.008 (−0.02, 0.002)	0.10	0.11	—	—	—
	Gait speed	−0.11 (−0.16, −0.07)	<0.001	0.09	−0.08 (−0.12, −0.03)	0.001	0.13	−0.08 (−0.13, −0.03) ^b^	0.001	0.19
*OCL*	HGS	0.002 (0, 0.004)	0.01	0.02	0.001 (−0.001, 0.003)	0.29	0.05	—	—	—
	ALM/height^2^	0.02 (−0.01, 0.02)	0.89	−0.003	−0.007 (−0.02, 0.007)	0.31	0.05	—	—	—
	Gait speed	0.06 (0.002, 0.12)	0.04	0.01	0.02 (−0.05, 0.09)	0.54	0.04	—	—	—
*OBK*	HGS	−0.004 (−0.005, −0.002)	<0.001	0.06	−0.002 (−0.004, 0)	0.04	0.13	—	—	—
	ALM/height^2^	−0.01 (−0.03, 0.001)	0.06	0.009	^#^	1.00	0.12	—	—	—
	Gait speed	−0.14 (−0.2, −0.08)	<0.001	0.06	−0.07 (−0.13, 0.001)	0.06	0.14	—	—	—
*OCF*	HGS	0.03 (0.02, 0.04)	<0.001	0.11	0.02 (0.008, 0.03)	0.001	0.20	0.02 (0.004, 0.03) ^c^	0.007	0.22
	ALM/height^2^	0.12 (0.03, 0.21)	0.01	0.02	0.02 (−0.07, 0.11)	0.69	0.16	-	-	-
	Gait speed	1.21 (0.80, 1.62)	<0.001	0.11	0.70 (0.27, 1.14)	0.002	0.19	0.52 (0.08, 0.96) ^c^	0.02	0.21

a HGS adjusted for age; gait speed (GS) adjusted for age and mobility; b. GS adjusted for age, BMI, mobility, smoking status; c. HGS and GS adjusted for age, education, and mobility. Other tested confounders were not significant and were excluded from the models.

HGS: handgrip strength (kg); ALM: relative appendicular lean mass (kg/m^2^);

DET: the detection task (log10 milliseconds) measuring psychomotor function; IDN: the identification task (log10 milliseconds) measuring attention; OCL: the one-card learning task (arcsine square root proportion correct) measuring memory/learning; OBK: one-back task (log10 milliseconds) measuring working memory; OCF: overall cognitive function (unitless). 95%CI: 95% confidence interval

-: muscle parameters do not contribute to the best-fitted models.

*: age, education, marital status, mobility, BMI, and smoking status contributed to the model.

^#^: The number was too small to be reported.

**Table 4:** Multivariable linear regression models (model 4) for final best-predicting DET, IDN and OCF.

		Coefficient B (95%CI)	p value	Adj. *R*^2^
DET				
			<0.001	0.13
	HGS	−0.003 (−0.005, −0.002)	<0.001	
	GS	−0.12 (−0.18, −0.05)	0.001	
	Constants	2.66 (2.59, 2.71)	<0.001	
IDN			<0.001	0.19
	GS	−0.08 (−0.13, −0.03)	0.001	
	Age	0.001 (0, 0.003)	0.02	
	BMI	−0.003 (−0.006, −0.001)	0.001	
	Physical activity	0.03 (0.01, 0.05)	0.002	
	Smoking	−0.07 (−0.12, −0.02)	0.008	
	Constants	2.74 (2.61, 2.87)	<0.001	
OCF			<0.001	0.22
	HGS	0.02 (0.003,0.03)	0.02	
	GS	0.49 (0.05,0.93)	0.03	
	Age	−0.02 (−0.03, −0.01)	<0.001	
	Secondary education completed	0.20 (0.03, 0.37)	0.03	
	Constants	0.73 (−0.31, 1.78)	0.17	

HGS: handgrip strength (kg); ALM: relative appendicular lean mass (kg/m^2^); GS: gait speed, measured as walking speed over 4 meters (m/s); DET: the detection task (log10 milliseconds) measuring psychomotor function; IDN: the identification task (log10 milliseconds) measuring attention; OCF: overall cognitive function (unitless); 95% confidence interval.

Handgrip strength, ALM/height^2^, and gait speed were all positively associated with IDN (model 1). After adjusting for age, the association of IDN with ALM/height^2^ was attenuated, but the association with HGS and gait speed persisted (model 2). The best fit model showed that for every 1.0 m/s increase in gait speed, IND decreased by 0.08 after adjusting for age, BMI, mobility and smoking status (model 3). HGS did not contribute to the final model (Table 3).

There were positive associations of HGS and gait speed with OCL and OBK (model 1, Table 3); the associations with OCL were attenuated after adjusting for age, but persisted with OBK (model 2). However, the associations of HGS and gait speed with OBK were explained by further adjustment for confounders (model 3). ALM/height^2^ was associated with OBK in model 1, but this association was attenuated after adjusting for age (model 2, Table 3).

### Sensitivity analysis

There were no differences in the patterns of associations described above, following exclusion of 12 outliers in sensitivity analyses (data not shown).

## Discussion

In this study, HGS was positively and independently associated with DET and OCF. In addition, gait speed was positively and independently associated with DET, IDN and OCF. No association was detected between lean mass and cognition, overall or in specific cognitive domains. Our study suggests that muscle strength or physical function is a better indication of cognitive function than lean mass, in older men.

We found that lower HGS was associated with poorer DET. Similar results were found in a study that compared HGS and psychomotor performance measures for elderly Caucasian women in the USA in both healthy (n = 19) and frail (n = 20) groups^40^. This study reported differences for both HGS and psychomotor performance between the healthy and frail groups. It is possible that a decline in muscle strength may share the same neurological mechanism underlying reduced reaction times in older people before cognitive decline begins in other domains. However, we did not detect associations between HGS and the other cognitive domains we assessed.

We found that slower gait speed was associated with reduced DET and IDN. This confirmed longitudinal findings from the Health, Ageing and Body Composition Study, which included 2,776 men and women aged 75–85 years^41^. In this study, gait speed was measured using walking speed test, while attention and psychomotor speed were assessed using the Digit Symbol Substitution Test (DSST) at baseline and after five years. Results showed that participants in the lowest quartile of gait speed were more likely to decline in DSST performance over five years, indicating that gait speed predicts decline in specific cognitive domains (attention and psychomotor speed) in the elderly^41^. It is relevant to note that the DSST involves paper and pencil and requires participants to copy as many novel symbols, corresponding to numbers, as possible in 1.5 minutes, while our task was computer based and required participants to respond to card stimuli as accurately and fast as possible. The decline in psychomotor function and attention could be due to changes in the white matter and hippocampus volume, which are consistently present in participants with slow gait speed and reduced executive and psychomotor function^42^. Our results also broadly agree with findings from a 6-year follow-up study of 2,654 men and women aged 60 to over 90 years that detected an association between gait speed and cognition including processing speed, executive function and verbal memory^42^.

Our study confirmed that low HGS was associated with poor OCF, consistent with previous research. Abellan van Kan *et al*. used the Short Portable Mental Status Questionnaire to measure general cognition and HGS to measure muscle strength in 3025 community-dwelling French women aged ≥75 years; their results indicated that lower HGS was associated with cognitive impairment^43^. The explanation could be that HGS decline is a marker of reduced physical health in the elderly, and associated with frailty, comorbid disease and mortality, whilst at the same time contributing to cognitive decline^5,7,44^. We found that slow gait speed was associated with poor OCF. This is consistent with the results of a study in Hong Kong, which examined the association between cognitive function and physical performance, measured using a 6-metre walking test, in 4,000 community-recruited Chinese men and women. The cognitively impaired group had poorer performance in gait speed tests than the non-cognitively impaired control group^45^.

We found that HGS and gait speed, but not lean mass, were associated with OCL and OBK, but that these associations were explained by age or other confounders. Therefore, this study does not support an association between muscle strength, physical performance, or lean mass with recognition memory/learning or working memory. However, to our knowledge, no studies have investigated the associations between muscle parameters and learning/memory to date. In addition, we have no evidence from our study that lean mass was associated with overall cognition. In 2018, van Dam *et al*.^46^ reported an association between cognitive functioning and muscle strength and lean mass in older patients in hospital (n = 378, 49.3% female, aged>70 years). Low cognitive function was assessed using Six-Item Cognitive Impairment Test (short questionnaire), muscle mass parameters (including appendicular lean mass) using direct segmental multifrequency bioelectrical impedance analysis (BIA), and muscle strength by HGS. At admission, lower cognitive functioning was associated with lower HGS and ALM. To note, differences in the participant characteristics (hospitalised vs general population), assessment tools for muscle (BIA vs DXA) and cognitive function (questionnaires vs computer-based), might have contributed to inconsistencies with our results.

Our study systematically considered potential confounders that have been examined previously. We found that age, BMI, self-reported levels of physical activity and smoking were associated IDN independent of GS, while age and education were associated with OCF independent of HGS and gait speed. Smoking, alcohol use, physical activity^25–27^ and education^25–27^ are known to be associated with both muscle health and cognition. Marital status in older participants is a marker for social isolation, loneliness and low levels of physical activity (device measured objectively)^28^. Divorced and widowed older adults are particularly at risk of developing negative health outcomes, such as frailty and depression^29^. A longitudinal study in the USA aimed to investigate marital status and cognitive impairment and included 7508 participants aged ≥65 years^47^. This study found that, compared to married counterparts, divorced and widowed elders had higher risk of developing dementia and non-demented cognitive impairment, as well as impairment in memory, orientation and executive function, while never married elders had higher risk of impairment in memory and orientation. A longitudinal study in Korea investigated the association between HGS and risk of cognitive impairment in 544 older women aged over 65 years^7^. Cognitive impairment was identified using Korean Mini-mental State Examination (K-MMSE). The finding was that HGS was associated with risk of cognitive impairment among obese women only.

Consistent with our results, previous longitudinal studies have found that slow gait speed predicts dementia independent of muscle mass, and a decline in gait speed also predicts the development of MCI^48,49^. Our HGS data support the contention that measures of muscle strength are more clinically relevant than lean mass for indicating poor cognition in older adults^50^. This is in agreement with the view of the revised definition of sarcopenia, the European Working Group on Sarcopenia in Older People 2 (EWGSOP2),  that muscle strength is a better predictor of negative health outcomes than muscle mass^51^. In a quantitative review, it was postulated that loss of muscle strength is a more consistent risk for disability and death than loss of muscle mass^52^; earlier articles had reported loss of strength occurred 2–5 times faster than loss of mass^52^. This may explain why our cross-sectional study suggests a parallel decline in cognition and muscle strength but not in lean mass. However, in a Belgian study of men and women aged 60–80 years, differences in HGS were found between the group with MCI and the normal group; no differences were found in physical performance (gait speed and balance measured by Short Physical Performance Battery) or body composition (muscle and fat mass)^53^.

Our study indicated that muscle strength and physical performance are better indicators for poor cognitive function, overall, and in some specific domains. There is a non-linear relationship between muscle mass and strength; muscle strength declines more rapidly with age than does muscle mass and this might reflect the importance of neuromuscular decline^23,54^. More recently, the EWGSOP 2^13^ placed more emphasis on reduced muscle strength rather than muscle mass. Age-related loss of muscle strength and power appear to be more useful for indicating the risk of physical disability^55^. We could speculate that there is a redundancy in muscle mass that allows for preservation of physical performance despite age-related atrophy, but this is beyond the scope of our data set.

We found that gait speed was a stronger indicator than HGS of IDN, DET and OCF. A recent Korean study involving older adults (70–84 years) reported that sarcopenia and gait speed were associated with information processing and executive function in men, but only gait speed was associated with impairment in those domains in women. The authors concluded that these associations were driven mainly by gait speed^56^.

A strength of our study is that participants were selected at random from the general population rather than on the basis of disease. However, ability to provide informed consent was an exclusion criterion; thus, individuals with severe cognitive impairment or dementia were not included in the study. As we included men only, and the sample was mainly Caucasian, our conclusions may not be generalisable to other populations. As this was a cross-sectional study, we cannot determine causality. We recognise the limitation of using lean mass measured by DXA as a surrogate measure of muscle mass, as DXA does not assess muscle quality or intramuscular fat that may be important in the muscle-cognition relationship. For example, fat infiltration, lean tissue thickness and hydration may not have been captured by DXA. HGS was measured using an electronic device that provides systematically lower strength measures than those reported for the Jamar manual dynamometer. Furthermore, we acknowledge that an individual’s cognitive function might have affected their physical performance in assessment tasks. As only four cognitive domains were tested, the results should not be generalised to other cognitive domains that we did not assess. As in all observational studies, confounding may not have been adequately accounted for, thus we cannot discount the possibility of residual confounding.

In conclusion, poorer cognitive function, especially in DET and IDN, was associated with lower muscle strength and poorer physical performance in the men in our study. This finding adds to the growing body of evidence that skeletal muscle and cognitive decline share common pathological pathways and that skeletal muscle might be a modifiable risk factor for cognitive impairment. Cognitive decline in tandem with loss of muscle strength and function places elderly people at increased risk of personal injury, poor mobility and loss of independence. Prospective epidemiological studies are warranted, and could include brain imaging to detect underlying common mechanisms for concomitant changes in brain and muscle.

## Data Availability

The datasets generated during and/or analysed during the current study are available from the corresponding author on reasonable request.
